# A Way of Bionic Control Based on EI, EMG, and FMG Signals

**DOI:** 10.3390/s22010152

**Published:** 2021-12-27

**Authors:** Andrey Briko, Vladislava Kapravchuk, Alexander Kobelev, Ahmad Hammoud, Steffen Leonhardt, Chuong Ngo, Yury Gulyaev, Sergey Shchukin

**Affiliations:** 1 Department of Medical and Technical Information Technology, Bauman Moscow State Technical University, 105005 Moscow, Russia; 9784882@mail.ru (V.K.); ak.mail.ru@gmail.com (A.K.); Hammoud@bmstu.ru (A.H.); schookin@bmstu.ru (S.S.); 2 Medical Information Technology, RWTH Aachen University, 52074 Aachen, Germany; leonhardt@hia.rwth-aachen.de (S.L.); ngo@hia.rwth-aachen.de (C.N.); 3 Kotelnikov Institute of Radioengineering and Electronics (IRE) of Russian Academy of Sciences, 125009 Moscow, Russia; gulyaev@cplire.ru

**Keywords:** electrical impedance, electromyogram, force myogram, sensor system, simultaneous acquisition, neuromuscular interface, prosthesis, orthosis

## Abstract

Creating highly functional prosthetic, orthotic, and rehabilitation devices is a socially relevant scientific and engineering task. Currently, certain constraints hamper the development of such devices. The primary constraint is the lack of an intuitive and reliable control interface working between the organism and the actuator. The critical point in developing these devices and systems is determining the type and parameters of movements based on control signals recorded on an extremity. In the study, we investigate the simultaneous acquisition of electric impedance (EI), electromyography (EMG), and force myography (FMG) signals during basic wrist movements: grasping, flexion/extension, and rotation. For investigation, a laboratory instrumentation and software test setup were made for registering signals and collecting data. The analysis of the acquired signals revealed that the EI signals in conjunction with the analysis of EMG and FMG signals could potentially be highly informative in anthropomorphic control systems. The study results confirm that the comprehensive real-time analysis of EI, EMG, and FMG signals potentially allows implementing the method of anthropomorphic and proportional control with an acceptable delay.

## 1. Introduction

The human arm is an incredibly complicated tool capable of performing many actions, including fine movements, due to a large number of degrees of freedom [1]. The functionality of arms is used for almost all daily activities such as eating, dressing, personal activities, and social interactions. The loss of movement of an upper extremity drastically worsens the quality of life [2].

Creating highly functional prosthetic [3,4,5,6,7], orthotic [8], and rehabilitation [9] devices is a socially relevant scientific and engineering task since it allows bringing patients back to active life by partial restoration of the lost motor functions of an extremity and reducing rehabilitation time.

However, despite the last 50 years of technological progress, the development of bionic devices still faces certain hurdles. The main hurdle rendering the devices far from satisfactory for end-users is the lack of an intuitive and reliable control interface [9,10,11], allowing to overcome the problem of replacing an upper extremity [12]. The high functionality and accuracy of modern actuators [13] are limited by the capabilities of the existing bionic control methods. The current control methods, for example, the methods based on individual signals obtained from one physical method, do not give detailed information about the biomechanical characteristics of muscle activity. So, these methods can only partially fulfill the requirements for anthropomorphic control, including movements of the copying type [13,14], force proportionality [13,15], combined movements, and the control delay of less than 150 ms [16,17].

So, the current trend of perfecting bionic control systems is a combined approach with measuring different types of signals [13,18,19,20]. Based on the literature review, we draw the following conclusions: surface EMG (sEMG) signal is still the most effective and popular method of evaluating muscle activity. However, the control based on this method is still not highly functional. On the other hand, combining the EMG signal with one or more signals registered using other physical methods allows radical improvement of control accuracy and reliability and gets closer to implementing anthropomorphic control. Except for EI, all other methods combined with EMG do not allow registering signals from one system. However, combining FMG with EI and EMG can be carried out without violating the rules of the conventional method of measurements from two channels from antagonistic muscles.

EMG-based methods measure the skin electrical activity occurring during repolarization of muscles during activation using electrodes [16,21,22]. The registered EMG signal is directly linked with physiological and anatomic factors such as the amount of active motor units, type of muscle fibers, diameter, depth and location of active fibers, and characteristics of the activation of motor units [23,24]. The control principle of most EMG-based commercially available systems is based on simple method of proportional control first proposed in the 20th century [1,25]. For better user experience, more movement types should be identified, so, a greater number of measurement channels used simultaneously is required [26,27,28,29]. High-density EMG methods are used, they allow obtaining depolarization signals caused by the activity of certain muscles [30,31].

The physical essence of the EI-based control method is that a system of current and potential electrodes is placed on the area to be investigated. A low-intensity current is passed between the current electrodes; the potential electrodes register the occurring voltage. The measured EI values carry information about the electrical properties of biological tissues at a probing depth. EI myography is based on non-invasive measurements linked with muscle activity during actions [32,33,34,35,36,37]. EI muscle tomography is a novel alley of research. This method allows identifying an adequate number of movement types based on classifiers using multi-channel electrode systems [38]. Unlike EMG and most conventional EI-based neurophysiological methods, the electrical activity of biological tissues is not investigated.

The FMG method is based on monitoring the changes in skin tension due to increased cross-section of an extremity occurring during muscle contraction [39,40,41]. FMG is widely used as an alternative to EMG in bionic device control [42,43] due to certain advantages. The advantages are the immunity to external electrical noise and perspiration [44], no requirements to the preliminary processing of the skin [45], milder requirements to software and hardware for registering and processing the signals [46], low sensor cost, and signal stability over time under static actions [47]. For most FMG applications, determining movement patterns requires two and more sensors to function [48].

**Figure 1:**
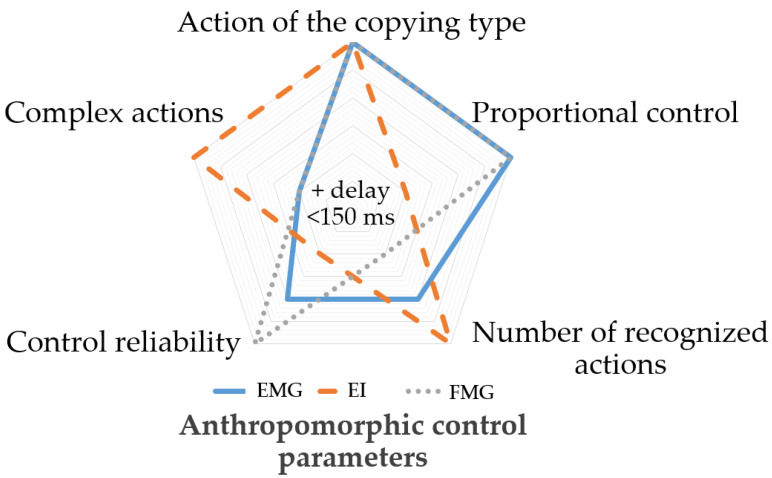
Anthropomorphic control parameters for EI, EMG, and FMG.

Based on our expertise, we complied the radar chart shown in Figure 1. The chart proves that combined use of EI, EMG, and FMG signals would allow overcoming many method-specific hurdles and improving control functionality and shows how each method is to be investigated to get the best result from combining. Thus, EI signal can yield biomechanical information about muscle contraction, EMG (electrical activity parameters) and FMG (pressing force and morphological changes).

In that sense, the EI signal combined with the EMG and FMG signal analysis can potentially be highly informative in anthropomorphic control systems. Moreover, this method would not require an increased number of measurement channels [49,50]. Thus, this paper investigates the simultaneously acquired EI, EMG, and FMG signals of wrist movement with a special sensory system to get closer to solving the relevant problem of anthropomorphic bionic control based on neuromuscular activity.

## 2. Materials and Methods

### 2.1. Equipment

#### 2.1.1. Simultaneous EI and EMG Acquisition

**Figure 2:**
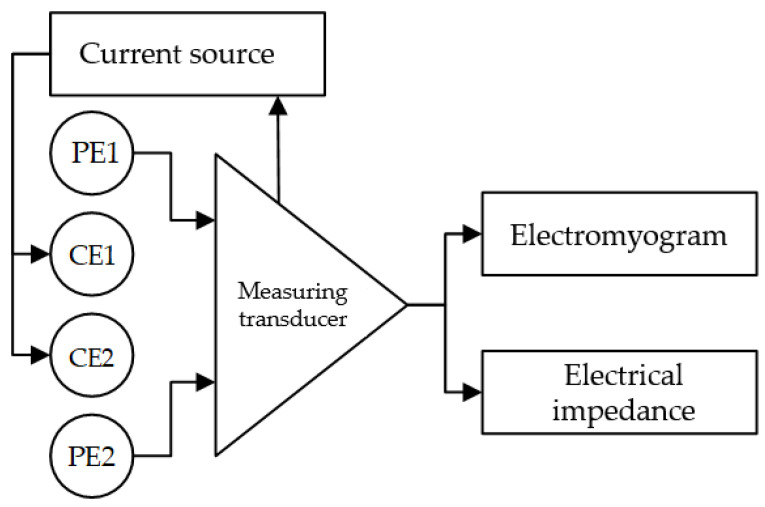
Simultaneous acquisition of EI and EMG signals (PE, potential electrode; CE, current electrode).

Simultaneous acquisition of EMG and EI signals from the same electrodes placed according to the tetrapolar measurement system, Figure 2, was carried out using a “Status-a” special laboratory dual-channel system produced by MTRT LLC, Russia.

The voltage at the potential electrodes is the algebraic sum of the EMG signal and the amplitude modulated (AM) voltage at the probing frequency occurring as the difference of potentials from a current source (EI signal). As these signals have different frequency ranges, they can be separated using bandpass filters (BPF). The EMG signal is separated from the EI signal with a BPF having a 50–400 Hz bandpass. The amplitude-modulated EI signal is separated from the EMG signal with a BPF with a 10 kHz–1 MHz bandpass. To extract the original EI signal from the 100 kHz AM signal, one has to perform amplitude demodulation using a phase-lock detector. For the phase lock detector, the reference carrier signal has the same frequency such as that of the current source. After additional amplification, signals from both channels are digitized. Thus, EMG and EI signal from the same channels are obtained.

In order to eliminate the cross-talk between two electric impedance channels, the channels are separated by phase. The current source of the first channel generates a sine signal, and the second channel generates a cosine signal. The “Status-A” system has a required sensitivity [51] and time resolution for the investigations conducted in this paper.

The signal-to-noise ratio (SNR) of the EMG signal was improved by increasing the EMG signal acquisition base, whereby potential electrodes were placed on the periphery, and the current electrodes were placed at the center. In terms of the EI signal, such placement is equivalent to the conventional placement of electrodes of the tetrapolar systems (current electrodes on the periphery, potential electrodes at the center) according to the reciprocity theorem. This approach was justified theoretically and experimentally in the previous works of the authors [52].

#### 2.1.2. Prototype of Sensor Systems

**Figure 3:**
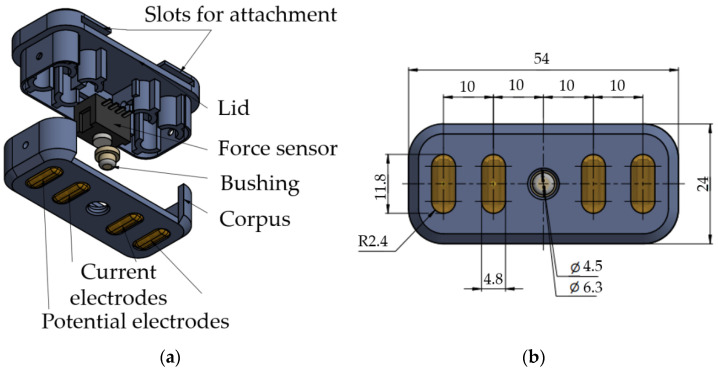
Developed sensor system: (**a**) components of the sensor system; (**b**) dimensions of the sensor system.

For simultaneous acquisition of the EMG, EI, and FMG signals, prototypes of special sensor systems, Figure 3, were made. The prototypes were developed based on the following criteria. First, the system size had to be similar to that of typical electrode systems (ES) in commercially available bioelectric devices. Second, two sensors should be positioned at the projection of antagonistic muscles of the forearm (according to the typical places of acquisition for the bioelectric arm prosthesis). Third, the system should be comfortable and reliably fixed for quality measurements.

The distance between electrodes was chosen to ensure the sufficiently small size of the sensor system and place the chosen sensor between them: 40 mm between the potential electrodes, 10 mm between the current electrodes. The electrodes were 12.5 mm long and 5 mm wide and had an elliptical shape. The electrodes’ material was steel coated with titanium nitride.

A Honeywell FSG15N1A force sensor was used as a force sensor sensitive enough to acquire FMG signals. The force sensor is installed in the dedicated slot in the lid of the sensor system and contacts the forearm skin via a special bushing. At the forearm, the sensor system is fixed using medical tourniquets passing through the case eyelets.

### 2.2. Isometric Grasping Stand

**Figure 4:**
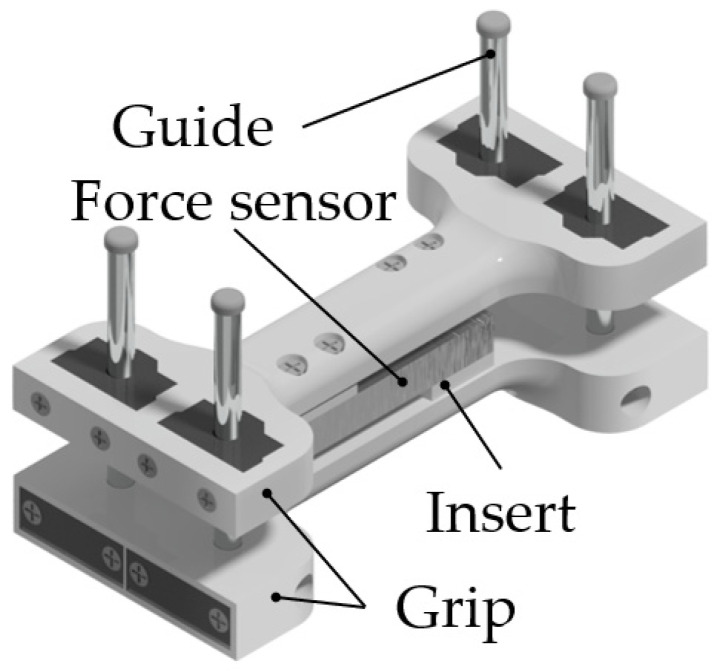
Isometric grasping stand.

A special isometric hand grasping force measuring stand, Figure 4, was used to identify the relationship between FMG signal and the grasping force for implementing proportional control. The stand was described in detail in the previous works of the author [53]. In the stand, the grasping force is transferred to a 40 kg (40 daN) strain gauge load cell via handles. The grasping force measurement error was 0.1 kg (0.1 daN). For the comfort of use, the handle size was chosen based on the wrist size of an adult person. The grasping width can be adjusted using variable-thickness inserts attached to the force sensor. Four guideways prevent the slanting of the stand.

### 2.3. A Laboratory Complex for Simultaneous Acquisition of EI, EMG, and FMG Signals

**Figure 5:**
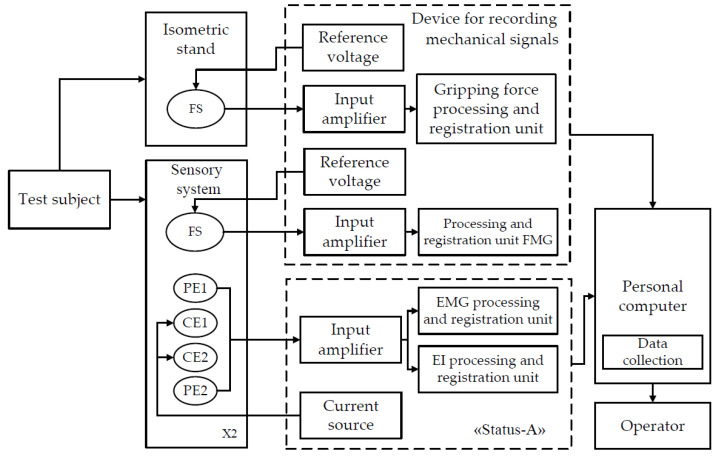
The layout of the laboratory hardware and software complex for simultaneous acquisition of EI, EMG, and FMG signals (FS, force sensor; PE, potential electrode; CE, current electrode).

For the study, a laboratory complex was developed and assembled. The complex comprises a device for bioelectric acquisition (EMG and EI signals) “Status-A”, an instrument for the acquisition of mechanical signals (FMG signal, grasping force of the isometric grasping stand), a sensor system, and a personal computer. Figure 5 shows the layout of the laboratory complex, and Table 1 presents its technical characteristics.

An instrumentation part of a Telereabos system (produced by scientific and medical firm MBN, Russia) was used to acquire mechanical signals. These instruments allow simultaneous acquisition of signals from up to four strain gauge force sensors with the required time resolution and sensitivity.

The devices were connected to a personal computer via the USB interface working in the VCP mode (Virtual COM Port). For simultaneous acquisition, visualization, and storing of the acquired signals, special software was used. MATLAB R2020b was used to process and analyze the acquired data.

### 2.4. Subjects

Seven men and women were chosen as test subjects (four men and three women). The subjects were 18–35 years old and had no diagnosed pathologies of the upper extremities. The circumference of the upper third of the forearm ranged from 0.2 m to 0.35 m.

The experiments have been conducted under the supervision of the Medical and Educational Center of Bauman Moscow State Technical University. The study followed the World Medical Association’s Declaration of Helsinki on Ethical Principles for Medical Research Involving Humans Subjects. All patients provided written consent before they participated in the study.

### 2.5. Experiments

#### 2.5.1. The Basic Wristed Movements Considered

**Figure 6:**
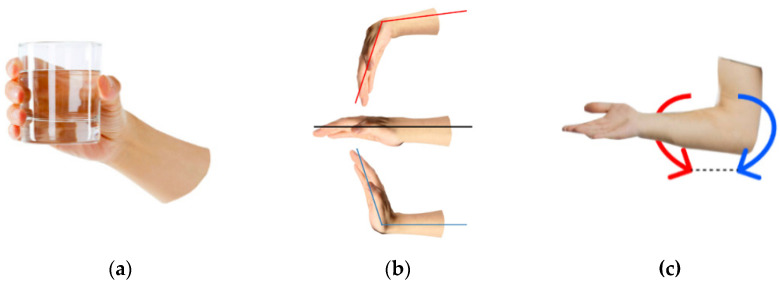
Basic wrist movements: (**a**) grasping, (**b**) flexion/extension, and (**c**) rotation.

The most common wrist movement in regular life is grasping; its types can be classified either based on the shapes of the grasped objects [54] or by grasping tasks ranging in force and accuracy [55]. The most functionally required wrist grasping types are the tip (closed) grasping and palm (open) grasping, Figure 6a. As tip and palm grasping are mechanically similar, the latter type will be considered in the study as the base movement.

In most bionic devices, wrist flexion/extension, Figure 6b, is not implemented due to technical difficulty. So, the wrist flexion angle has to be changed using the elbow joint, which does not satisfy the anthropomorphic principles. Adding this type of action would greatly widen the daily use of prosthetic devices. The rotation, Figure 6c, has to be implemented due to the need to control the wrist orientation and the complexity of performing this action artificially with the actuator without this functionality. Wrist rotation allows it to assume the optimal pose. Also, it plays a significant part in performing all wrist functions, including vocation-related movements. So, the study considered the basic wrist movements of grasping, flexion/extension and rotation (pronation/supination).

#### 2.5.2. Location of Sensor Systems

**Figure 7:**
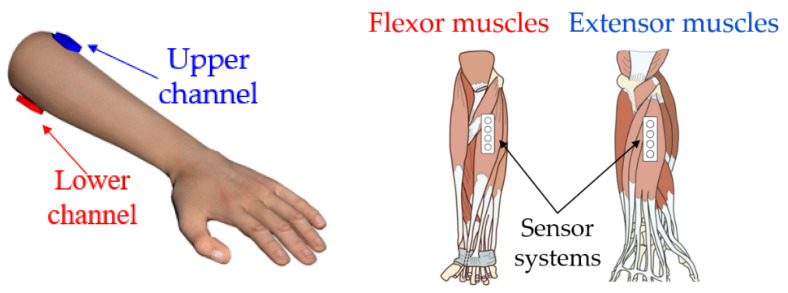
The location of the sensors systems in the projection of the muscles.

For simultaneous analysis of signals during basic wrist movements to solve the task of bionic control, two sensor systems were used for acquisition (two channels were used for signal acquisition). For signal acquisition, the sensor systems were placed at the upper third of the forearm in the projection of the antagonistic muscles. The upper channel was placed on extensor muscles (musculus extensor carpi ulnaris), and the lower channel was placed on flexor muscles (musculus flexor carpi radialis), Figure 7, according to the conventional principles of construction bioelectrical forearm prosthetics.

Before placement of the sensor system, the skin was processed with the Grass^®^ EC3^®^ conductive adhesive gel (USA) and the high-conductivity spray “Unispray” (Geltek, Russia). The sensor systems were attached to the forearm with a small pressing load adjusted using the medical tourniquets. The pressing force ranged from 0.2 daN to 0.4 daN and was determined based on the following criteria: a patient is comfortable, the contact is stable, the EI signal change during actions is reproducible and articulated [56].

#### 2.5.3. Experiments Protocol

During the study, the patients performed the aforementioned wrist actions (grasping, flexion/extension, and rotation (pronation/supination)). Each action was carried out without force and with maximum amplitude. In the experiments with the isometric grasping stand, the patients iteratively performed wrist grasping with increasing force. The EI, EMG, and FMG signals from both sensor systems were acquired in the monitoring mode. The measurements started after stabilization of the EI signal with full relaxation of the wrist. If the actions were not performed well enough, these measurements would be removed from further analysis. The patients were in a vertical position (sitting on a chair) and rested on the elbow. For each cycle, the measurements did not last longer than 10 min.

## 3. Results

### 3.1. Simultaneous Analysis of EI and EMG Signals during Basic Wrist Movements

**Figure 8:**
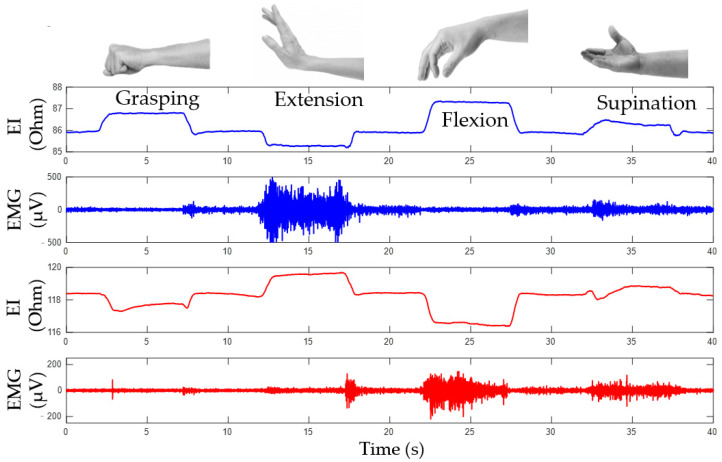
Simultaneous acquisition of EI and EMG signals during performing of movements.

Based on the conducted investigations, the study proposes that proportional force control can be implemented based on the analysis of amplitude parameters of EMG signals, while the type of the performed movement can be determined based on the analysis of the change of the EI signals. Figure 8 shows the EI and EMG signals during the following movements: grasping, flexion, extension, and supination.

#### 3.1.1. Determining Movement Type Based on EI Signal

**Figure 9:**
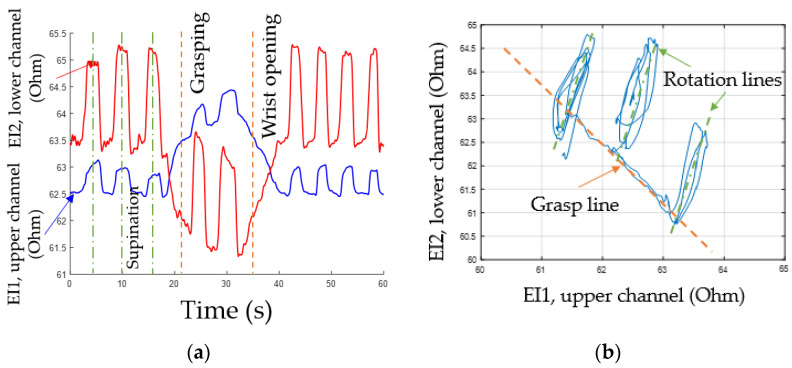
Analysis of EI signals: (**a**) experimental signal data during movements, (**b**) phase diagram.

EI signals from both channels are phase-aligned during rotation and have inverse phases during grasping and flexion/extension as demonstrated by lines for articulated phases of movements, Figure 9a. So, a special method of signal representation can be used. The main idea is to represent the acquired EI signals from antagonistic muscles on a diagram where the signals from the upper ES are plotted on the horizontal axis, while the signals from the lower ES are plotted on the vertical axis, Figure 9b.

During “grasping” or “flexion” movements, the movement of the point of current value happens along the line dubbed the “grasp line.” During rotation, the point’s movement can also be represented as a “rotation line” positioned at a certain angle relative to the “grasp line”; the angle is characteristic for each test subject. The state characterizing the full movement is described by extreme points on the phase diagrams. The lower right point corresponds to the grasping line, while the upper right point corresponds to the rotation line. Thus, by the movement of the point of instantaneous values of the signal along the lines of grasping and rotation, it is possible to determine the type and intensity of a movement.

#### 3.1.2. Determining Movement Intensity Based on EMG Signal

The intensity of muscle contractions depends on the motor units’ recruitment and increasing their excitation frequency [23]. The conventional approach to bionic control based on proportional control is based on the time-domain evaluation of EMG signal amplitude characteristics. However, this approach is incapable of controlling several different movements [16]. Due to the simplicity of calculations and low time delay, this approach is still popular.

**Figure 10:**
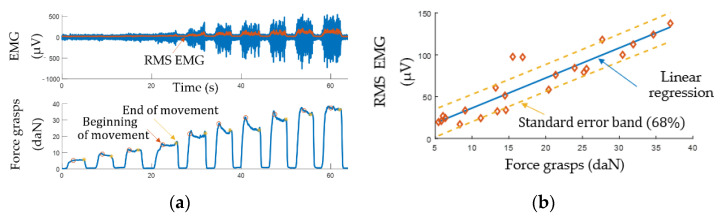
EMG signal analysis: (**a**) experimental data; (**b**) RMS EMG vs. force.

Figure 10 shows an experimental relationship of EMG vs. grasping force with which the test subject grasped the isometric stand with a different force. The measurements were made with the lower channel. The EMG signal was plotted as the 150 ms rolling RMS. The measurement results yielded a well-known [57], relationship of EMG vs. force which allows building a regression model for implementing proportional control.

### 3.2. Simultaneous Analysis of EI and EMG Signals during Basic Wrist Movements

**Figure 11:**
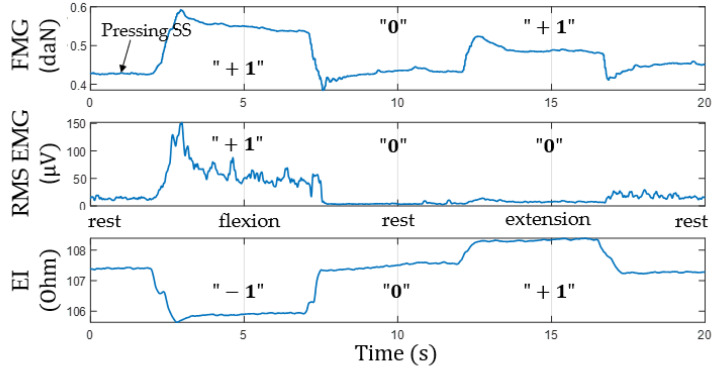
Simultaneous acquisition of EI, EMG, and FMG signals from extensor muscles during flexion/extension (“+1”, signal increase; “0”, no change; “−1”, signal decrease; SS, sensor system).

Figure 11 shows an example of signals acquired from the lower channel (pressing sensory system located at the projection of the forearm extensor muscles) during flexion/extension. The plots show that EI and EMG signals occurring during movements are out of phase. We believe that the phase shift of the EI signal is also related to physiological reasons besides morphological changes related to the increased pressing of the sensory system to the forearm.

**Figure 12:**
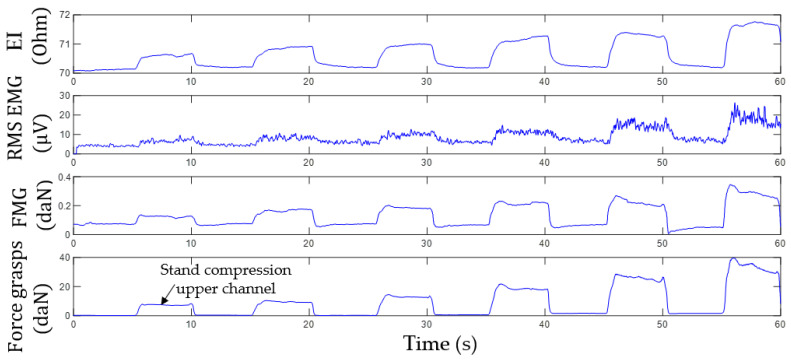
Time histories of EI, EMG, and FMG signals during grasping the isometric stand with variable force. The sensor system is placed at the projection of extensor muscles.

Figure 12 shows an example of the acquired signals from the upper channel (the sensor system placed at the projection of the forearm extensor muscles) during variable-intensity grasping using the isometric grasping stand.

**Figure 13:**
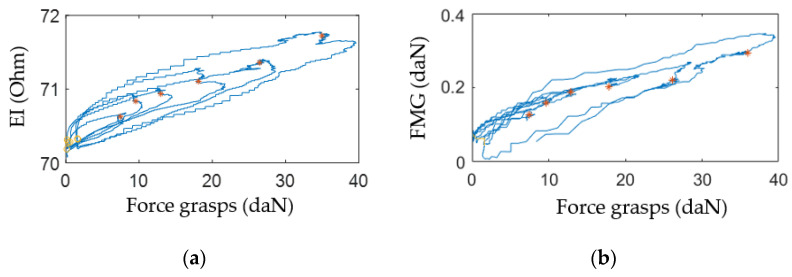
Change of EI (a) and FMG (b) signals depending on force grasps of the isometric grasping stand recorded with the sensor system placed at the projection of the extensor muscles.

The plots, Figure 13, built based on experimental data, Figure 12, indicate that the change of EI and FMG signals grows with the increase of the grasping force. This fact proves that proportional control can also be implemented using these signals.

The experiments established that high-quality reproducible EI and FMG signals cannot be obtained if the sensor system is misaligned. So, the sensor system must be located on the center of the belly of a contracting muscle and properly attached to the arm.

Finally, the obtained relationships were analyzed qualitatively. Table 2 shows the characteristic change patterns for the EI, EMG, and FMG signals from both channels during basic movements. The amplitude increase, decrease, and no amplitude change was marked as “+1”, “−1”, and “0”, accordingly. For the EMG signal, the standard deviation with the time window of 100 ms was analyzed.

## 4. Discussion

Currently, the control complexity remains the most severe challenge in the task of creating multi-DOF bionic devices for upper extremities. For example, the functionality of a purely EMG-based bioelectric prosthesis is limited by the number of independent EMG signals that can be acquired from the remaining extremity [6]. Nowadays, machine learning methods are widely used for extracting control information from a larger number of channels [28,29]. Such approaches are based on classification of signals where a set of informative EMG signal features corresponds to a set of movements being performed.

As far as the anthropomorphic control is concerned [14], the control functionality for bionic devices should be complemented with proportional control for each movement based on the estimate of amplitude parameters of control signals [13,58]. This requirement also applies to the analysis of combined movements [29]. In this case, the EMG-based classification only allows performing one movement at a time without independent speed and force control of both the individual and the combined movements [28].

To make the prosthesis control more comfortable, the current trends of perfecting bionic control systems are focused on combining the EMG signal with one or more signals based on a different physical method [28,43]. This combination of signals allows for significant improvement of control accuracy and reliability, as well as enriching the device functionality in terms of proportional and combined control. As mentioned above, this study investigates the possibility of the simultaneous analysis of EI, EMG, and FMG signals from two channels to implement anthropomorphic bionic control for the basic wrist movements: grasping, flexion/extension, and rotation.

### 4.1. Combining EMG, EI, and FMG Signals

The analysis of the results of investigating the EI, EMG, and FMG occurring during basic wrist movement acquired using the developed sensor system prototype indicated that using the FMG signal improves the descriptiveness during the analysis of the movement type and intensity [59,60]. In terms of evaluating movement intensity, EMG allows evaluating movements with large and medium intensity, while the FMG signal is more sensitive to small intensities. The FMG signal in conjunction with the EMG signal allows detecting a movement performed by the muscles groups on the side opposite to the side where the sensor system was located, which gives more information for the movement type identification, Table 2.

**Figure 14:**
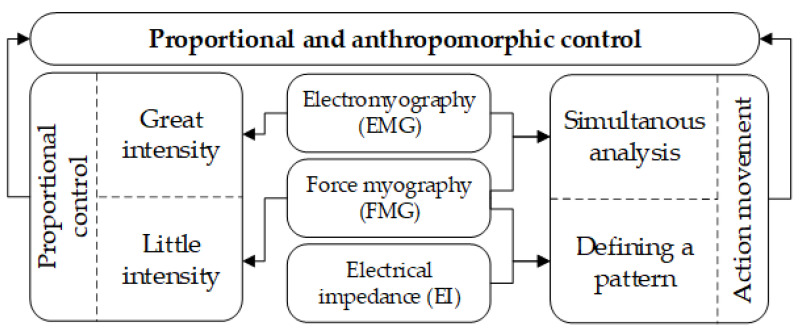
Scheme of simultaneous acquisition of EI, EMG, and FMG signals.

Thus, determining the movement type can be carried out based on EI signal patterns or based on the simultaneous analysis of EMG and FMG signals. The beginning of a movement can be registered based on the EMG signal. This principle of the analysis for proportional and anthropomorphic control is shown on a diagram for simultaneous analysis of EI, EMG and FMG signals, Figure 14.

The study established that flexion, extension, and rotation (rotation corresponded to supination since the initial wrist position corresponded to pronation) can be determined unambiguously. However, the patterns for grasping and flexion may coincide since the same muscle groups take part in these movement types. In this case, the absolute change of EI should be analyzed. During grasping, the signal changes less than during flexion since grasping mostly involves deep muscles. The muscles responsible for flexion/extension are closer to the hand surface and basically cover the muscles responsible for grasping and rotation. It means that EI and EMG signals will always be more articulated during flexion or extension, so their amplitude will be greater than that of the signals related to the grasping movement.

To improve the stability of determining movement type based on EMG signal, one can identify the instances of maximum movement. These instances correspond to the grasp line end points and rotation on the EI phase diagram. This operation will allow removing the phase diagram points displaced as a result of a low-frequency EI signal trend or movement artifacts from the calculations.

### 4.2. Limitations

In this study, the signals were acquired only from a small number of healthy volunteers without pathologies of the upper extremities. The proposed approach requires careful positioning of sensor systems with respect to the forearm muscles working during movements so that the signal’s analyzed pattern and amplitude characteristics would be articulate enough. The volunteers with a larger skin and fat layer indicated the amplitude smaller than that of the volunteers with a thinner skin and fat layer. Thus, the task of individual selection of the sensor system dimensions for improving the method reliability is relevant.

To integrate the proposed method in wearables, the signal acquisition hardware should be wireless since the movement of subjects during acquisition sometimes affects the signal stability due to the connecting wires. In this study, a spray was used for decreasing the “electrode-skin” resistance. However, the spray would dry out during longitudinal operation, significantly affecting the quality of EMG and EI signal acquisition.

### 4.3. Future Work

Future work should focus on developing algorithms and software for the method of anthropomorphic and proportional control based on simultaneous analysis of EI, EMG, and FMG signals, as well as verification of the accuracy of automatic classification and the analysis of parameters of the performed movements based on test sets of signals. To determine the severity of the aforementioned constraints for this method, the longitudinal measurement samples should be built based on data from subjects with different anthropomorphic features, both healthy and with partial loss of movement of the upper extremity, including amputees. To determine the possibility of analyzing more wrist movements and softening the requirements for accurate positioning of sensor systems (which is crucial to the patient comfort), we plan to conduct more studies with a larger number of sensor systems and different positioning on the forearm.

## 5. Conclusions

This study proposed a way of bionic control of devices based on EI, EMG, and FMG signals. The essence of the method is simultaneous acquisition and analysis of signals from two channels from antagonistic forearms muscles during basic wrist movements of grasping, flexion/extension, and rotation. For the study, a laboratory hardware and software complex with sensor system prototypes for registration and acquisition of signals.

The study revealed that the simultaneous real-time analysis of the acquired signals could potentially allow implementing the method of proportional and anthropomorphic control with an acceptable delay. The movement type can be identified based on the analysis of EI signal patterns and the simultaneous analysis of EMG and FMG signals. EMG signal can be used for movement with large and medium intensity, and the FMG signal can be used for movements with small intensity.

Integrating the proposed EI, EMG, and FMG-based method in wearables is a promising research direction. However, additional experiments should be carried out with more test subjects having different anthropometric features. Also, experiments with complex movements should be carried out. Nevertheless, the number and placement of sensor systems correspond to the conventional principles for the existing bioelectric prosthetics. So, this method can be used as an alternative to the existing ones. Finally, the study described the main limitations of this method.

## Figures and Tables

**Table 1:** Technical parameters of the laboratory complex.

Parameter		Value	
Number of channels		2	
Sampling frequency		1 kHz	
Types of acquired signals	EI	FMG	EMG
Maximum amplitude	300 Ω	10 daN	3 mV
Measurement error	10 mΩ	0.01 daN	10 μV
Signal frequency range	0–40 Hz	0–10 Hz	50–500 Hz
Probing current amplitude	5 mA	-	-
Probing current frequency	75 kHz	-	-

**Table 2:** Determining movement type (“+1”, increasing signal; “0”, no change; “−1”, decreasing signal).

		Movement Type				
Channel	Signal	Opening	Grasping	Flexion	Extension	Rotation
	EI	“0”	“+1”	“+1”	“−1”	“+1”
Upper	EMG	“0”	“0”/“+1”	“0”	“+1”	“+1”
	FMG	“0”	“+1”	“+1”	“+1”	“+1”
	EI	“0”	“−1”	“−1”	“+1”	“+1”
Lower	EMG	“0”	“0”/“+1”	“+1”	“0”	“+1”
	FMG	“0”	“+1”	“+1”	“+1”	“+1”

## Data Availability

The data presented in this study are available on request from the corresponding author.
